# Ovariotomy and Listerism

**Published:** 1890-01-25

**Authors:** 


### SURGERY.

### OVARIOTOMY AND LISTERISM.

Dr. G. Granville Bantock, surgeon to the Samaritan Free Hospital, publishes a paper in the December *Provincial Medical Journal*, headed "Table of 238 Cases of Ovariotomy," which carries his number of published cases up to 400. In 1881 there were only two surgeons, Mr. Lawson Tait and the writer, who ventured to perform ovariotomy without rigid observance of the Listerian method. But now it is rare to see the spray in use at all. The results in the Samaritan Free Hospital from April, 1885, to October, 1888, were, no mortality in a series of ninety cases. Under the Listerian method the mortality exceeded 12 per cent. For hands, instruments, and sponges, Dr. Bantock uses plain water and soap without any precautions to sterilise the water. The peritoneum is also washed out with the same water. Moreover, Dr. Bantock protests against the employment of any so-called germicide substance in flushing the peritoneum, for he believes that serious results may ensue from the use of carbolic acid or corrosive sublimate. The carbolic acid may be absorbed by the peritoneum, and some persons are peculiarly sensitive to any mercurial. Moreover, the author states that his patients recovered with less pyrexia than under the carbolic acid treatment. Now we have been much accustomed to attribute recent operative successes to the Listerian method, and failure to some fault in its application. Have we been wrong? is now the question which may be fairly asked. Dr. Bantock's success would seem to prove that "germs" may be dispensed with equally well by cleanliness as by the use of antiseptic sprays and dressings. We should be glad to see a Listerian reply to Dr. Bantock's recent paper under notice, and to his previous article on Listerism read before the Royal Medical and Chirurgical Society.

